# Successful adaptation of an initiative to reduce unnecessary antibiotics for acute respiratory infections across two Veteran Affairs ambulatory healthcare systems

**DOI:** 10.1017/ash.2024.357

**Published:** 2024-10-03

**Authors:** Morgan C. Johnson, Jessica G. Bennett, Milner B. Staub, Neena Thomas-Gosain

**Affiliations:** 1 Departments of Medicine, Pharmacy, and Geriatric Research, Education and Clinical Center, Veterans Affairs Tennessee Valley Healthcare System, Nashville, TN, USA; 2 Departments of Medicine and Pharmacy, Veterans Affairs Memphis Healthcare System, Memphis, TN, USA; 3 Division of Infectious Diseases, Vanderbilt University Medical Center, Nashville, TN, USA; 4 Division of Infectious Diseases, University of Colorado Anschutz Medical Center, Aurora, CO, USA

## Background

In the Veteran Affairs system, antibiotic prescribing for acute bronchitis (AUB) was previously reported in as high as 85% of visits.^1^ Provider audit-and-feedback (feedback) successfully reduces antibiotic prescribing for upper respiratory tract infection (URI) diagnoses like AUB but requires ongoing intervention to maintain results.^2–5^ Many antimicrobial stewardship programs (ASPs) struggle to implement, disseminate, and sustain feedback due to limited resources.^6^

VA Tennessee Valley Healthcare System (TVHS) adapted traditional feedback into a more resource-efficient model of targeted, quarterly feedback for high prescribers, successfully reducing and maintaining lower antibiotic prescribing for AUB and URIs, not otherwise specified.^3,7^ Here, we describe adaptation and implementation of this intervention at Lieutenant Colonel Luke Weathers, Jr. VA (Memphis) using the Adaptome sources of intervention adaptation framework.^8^

## Methods

### Settings

TVHS has 18 community-based outpatient clinics (CBOCs), serving >105,000 Veterans. TVHS intervention team included an infectious diseases (ID) physician and nurse practitioner, each dedicating 25%–50% full-time equivalent (FTE) to the project, and an ID pharmacist contributing 0.1 FTE.

Memphis has 11 CBOCs serving ∼47,000 Veterans. The Memphis team included an ID physician and three ID/ASP pharmacists dedicating 0.15 weekly FTE collectively and a support clinical pharmacist serving as liaison to clinic Medical Directors.

### Adaptation of existing intervention

The Adaptome platform was introduced as a conceptual model to collect, categorize and standardize measurement of adaptations of evidence-based practice implementation across systems.^9^ It includes a framework, used previously to describe the scaling-up^9^ and scaling-out^10^ of successful interventions, that lists potential sources of intervention adaptations organized into 5 crucial areas to consider when implementing an intervention: service setting, target audience, mode of delivery, culture context, and core intervention components (Supplemental Figure 1). Beginning February 2021, TVHS and Memphis teams met to identify differences between systems and utilized the Adaptome to guide assessment and adaptation of TVHS’s previously described multistep, targeted feedback intervention^3^ (Table 1).

**Table 1.** Areas for potential adaptation, adaptations made, and reason for adaptations guided by the Adaptome framework

Intervention	TVHS	Memphis	Adaptations made	Adaptome parameter	Why
**Preassessment**	Coordination e-mail with practice manager to schedule meetings with providers	Introduce initiative at ambulatory care meeting ahead of time; utilized academic detailer	More transparency in the initiative	Core component	Potential increased buy-in
	No materials provided ahead of survey	Patient education materials delivered ahead of interview (4/5 PACTS)	Patient education prior to survey	Core component	For provider review and availability
	No preview of survey questions	E-mailed survey ahead of time	Helped narrow discussion	Core component	Identified areas to focus on during interview, less momentary sway/off-topic discussion
	Original survey	Survey changes	Minor changes in questionnaire	Core component	Narrowed focus to just URI; added questions about point-of-care testing
**Interview**	In person with script, individual	Virtual focus groups	Virtual	Mode of delivery change and cultural adaptations	COVID-19
				Service setting change	Financial (limited FTE)
			Group	Mode of delivery change	Covid and staffing availability/time
	MD and NP (occasionally PharmD)	PharmDs and one MD	Provider vs non-provider interviewer	Service setting change	Staffing availability
	No grading, but performance reviewed	Subjective assessment of interviewee receptiveness; grading system and review of performance	Assessment of buy-in	Target audience adaptation	TVHS not getting buy-in from providers that continued to be high prescribers
**Report cards**	In-person feedback for first report and data compared to peers (all PCPs); e-mailed subsequent every month (at one point)	E-mailed after interview individual and specific clinic peers	Frequency	Service setting change	Staffing availability
	No supervisor involvement	Supervisors tagged on individual report cards	Leadership involvement	Cultural adaptations	Perception of supervisor involvement
	Monthly e-mails, all providers	Bi-annual e-mails, all providers	Frequency	Mode of delivery change	Monthly was ineffective at TVHS
	Monthly e-mails changed to only high prescribers	Quarterly e-mails, high prescribers, use of TVHS template	Frequency	Service setting change	Staffing availability and smaller system
	Monthly updates via peer-comparison retrospective feedback and education	Quarterly, semi-annual updates to ambulatory care staff meeting	Continued buy-in with PCPs	Mode of delivery change	Staffing availability, smaller system, and ineffective communication strategy at TVHS per read receipts
**Addressing high prescribers**	Alerted individual high prescribers, notified supervisor, and requested one-on-one guided chart review	No one-to-one feedback	No feedback at Memphis	Core component	Perception of feedback vs punishment
	Quarterly review of high prescribers				
**Sustainment**	Quarterly review of prescribers with notification to high prescribing individuals ***with an offered one-on-one chart review***	Quarterly review of prescribers with notification to high prescribing individuals	No one-to-one chart review offered at Memphis	Core component	Prospective feedback occurring already by ASP and academic detailer completing high prescriber visits
	Academic detailing not involved	Academic detailing pharmacist does one-on-one review for high prescribers	Utilization of academic detailing pharmacist	Service setting change	Academic detailer willing to assist with feedback

### Interventions and adaptations

Several adaptations stemmed from TVHS’s learned experience, prompting adaptations that improved efficiency and selecting for interventions with higher return on investment. Other adaptations arose from key differences in Memphis’s clinical context and culture, including: Memphis’s preexisting ASP relationship with and inclusion of supervisors in data reporting which was considered punitive at TVHS; the widespread use and acceptance of virtual meetings at Memphis due to COVID-19; and the significant reduction in available FTE at Memphis (Table 1).

The intervention was introduced to Memphis providers during primary care staff meeting in April 2021 followed by an invitation to a virtual group interview and a preinterview assessment survey. Interviews were performed with providers from the same clinic and questions tailored to the survey responses. Patient education materials on URIs were limited and therefore, provided to select CBOC sites (4/11 clinics) with more prescribers. The pharmacist liaison and primary care leadership contacted providers who did not initially respond to urge them to complete the survey and schedule interviews. Individual and clinic report cards (Supplemental Figure 2) and provider education were e-mailed to participants immediately following interviews and quarterly thereafter. Interviews were conducted from June through September 2021. Several adaptations were made in Memphis to the preassessment and interviews adapted from in-person, individual interviews to virtual group interviews with preassessment of provider attitudes and perceptions prior to initial interviews.

Memphis transitioned directly to quarterly targeted feedback to high prescribers, those with a prescribing rate > 25% with more than four encounters in the specified period, eliminating several feedback models deemed inefficient at TVHS. This cutoff was chosen to ensure that frequent over-prescribers were included, but infrequent prescribers were not unnecessarily chastised. However, exactly as done at TVHS, providers were contacted via e-mail regarding their performance and given the opportunity to meet one-on-one to discuss additional assistance from ASP and review specific cases.

### Outcome measures

The National VA Academic Detailing Database acute respiratory infection dashboard was used to identify AUB/URIs encounters and antibiotic prescribing. TVHS and Memphis manually chart reviewed all AUB/ URI encounters without associated antibiotic prescriptions to verify whether an antibiotic had been prescribed. Monthly percentage of AUB/URI visits with an antibiotic prescription were tabulated (Supplemental Table 1) and graphed (Supplemental Figures 3–4).

### Statistical analysis

Proportion AUB/URI encounters with antibiotic prescriptions was analyzed using Microsoft ® Excel (version 2302, Redmond, Washington) with QIMacros (version 2022.01, KnowWare International Inc., Denver, Colorado) to generate statistical process control P-charts. Two-sample proportions test with 95% confidence intervals (CIs) was used to compare pre- and postimplementation prescribing (STATA/MP 16.1, College Station, Texas). TVHS and Memphis Institutional Review Boards deemed the project quality improvement.

## Results

At Memphis, 33/48 (69%) providers completed presurveys and 41/48 (85%) participated in group interviews. From preimplementation (March 2020–March 2021) to postimplementation (October 2021–September 2022), Memphis antibiotic prescribing decreased from 33% to 15% (difference 17%; 95% CI, 7%–27%); TVHS sustained reduced antibiotic prescribing (Supplemental Table 1 and Supplemental Figures 3-3).

## Discussion

This study describes the process of successful adaptation and implementation from one VA to another of targeted, quarterly feedback to high antibiotic prescribers to reduce antibiotics for AUB/URIs. To the authors’ knowledge, this is the first study to use the Adaptome framework to describe the process of adapting and implementing a successful AS intervention across healthcare systems. Important to ASPs, it is imperative to anticipate mismatches in context, culture, and setting and plan for intervention adaptations to improve efficacy. These findings underscore the need for additional studies that assess methods for adapting successful interventions between ASP sites.

## Supporting information

Johnson et al. supplementary material 1 Johnson et al. supplementary material

Johnson et al. supplementary material 2 Johnson et al. supplementary material

## Supplementary material

To view supplementary material for this article, please visit https://doi.org/10.1017/ash.2024.357
