# Supplementary material for: Successful adaptation of an initiative to reduce unnecessary antibiotics for acute respiratory infections across two Veteran Affairs ambulatory healthcare systems
Source: Antimicrob Steward Healthc Epidemiol. 2024 Oct 3;4(1):e156. doi: 10.1017/ash.2024.357 (PMC11450663; doi:10.1017/ash.2024.357)
Supplement: Johnson et al. supplementary material 1 — Johnson et al. supplementary material [file S2732494X24003577sup001.docx]

**Supplemental Table 1: Counts of URI and AUB Encounters with Antibiotics and Total for Nashville and Memphis, January 2018 through September 2022**

| **Facility** |  | **Jan** | **Feb** | **Mar** | **Apr** | **May** | **Jun** | **Jul** | **Aug** | **Sept** | **Oct** | **Nov** | **Dec** |
| --- | --- | --- | --- | --- | --- | --- | --- | --- | --- | --- | --- | --- | --- |
| **2018** | | | | | | | | | | | | | |
| **Nash** | **Abx** | 103 | 72 | 54 | 57 | 36 | 31 | 25 | 36 | 38 | 51 | 75 | 89 |
|  | **Total** | 195 | 104 | 88 | 84 | 56 | 41 | 32 | 55 | 65 | 78 | 114 | 142 |
| **Mem** | **Abx** |  |  |  |  |  |  |  |  |  |  |  |  |
|  | **Total** |  |  |  |  |  |  |  |  |  |  |  |  |
| **2019** | | | | | | | | | | | | | |
| **Nash** | **Abx** | 115 | 76 | 44 | 28 | 28 | 18 | 25 | 15 | 18 | 26 | 33 | 34 |
|  | **Total** | 168 | 142 | 89 | 58 | 54 | 30 | 39 | 28 | 48 | 45 | 78 | 98 |
| **Mem** | **Abx** |  |  |  |  |  |  |  |  | 10 | 19 | 15 | 24 |
|  | **Total** |  |  |  |  |  |  |  |  | 21 | 32 | 37 | 57 |
| **2020** | | | | | | | | | | | | | |
| **Nash** | **Abx** | 29 | 17 | 15 | N/A | 3 | 2 | 4 | 5 | 4 | 10 | 6 | 1 |
|  | **Total** | 92 | 65 | 40 | N/A | 11 | 7 | 11 | 9 | 12 | 27 | 24 | 21 |
| **Mem** | **Abx** | 25 | 13 | 24 | 8 | 5 | 1 | 2 | 1 | 0 | 0 | 3 | 2 |
|  | **Total** | 64 | 41 | 76 | 19 | 13 | 5 | 7 | 5 | 5 | 6 | 9 | 7 |
| **2021** | | | | | | | | | | | | | |
| **Nash** | **Abx** | 4 | 2 | 5 | 6 | 4 | 7 | 11 | 12 | 7 | 3 | 9 | 10 |
|  | **Total** | 19 | 5 | 13 | 16 | 11 | 20 | 18 | 23 | 22 | 20 | 39 | 46 |
| **Mem** | **Abx** | 2 | 4 | 3 | 2 | 4 | 5 | 10 | 3 | 6 | 1 | 1 | 1 |
|  | **Total** | 5 | 4 | 7 | 5 | 9 | 11 | 15 | 14 | 11 | 7 | 10 | 23 |
| **2022** | | | | | | | | | | | | | |
| **Nash** | **Abx** | 8 | 3 | 9 | 8 | 12 | 4 | 5 | 6 | 8 |  |  |  |
|  | **Total** | 39 | 21 | 19 | 18 | 24 | 16 | 16 | 22 | 20 |  |  |  |
| **Mem** | **Abx** | 1 | 3 | 0 | 2 | 1 | 1 | 1 | 3 | 2 |  |  |  |
|  | **Total** | 10 | 7 | 2 | 4 | 8 | 7 | 6 | 9 | 17 |  |  |  |
|  |  |  |  |  |  |  |  |  |  |  |  |  |  |

Abbreviations:

Abx Antibiotics-- Encounters in which an antibiotic was given

Nash Nashville

Mem Memphis
