# Supplementary material for: Successful adaptation of an initiative to reduce unnecessary antibiotics for acute respiratory infections across two Veteran Affairs ambulatory healthcare systems
Source: Antimicrob Steward Healthc Epidemiol. 2024 Oct 3;4(1):e156. doi: 10.1017/ash.2024.357 (PMC11450663; doi:10.1017/ash.2024.357)
Supplement: Johnson et al. supplementary material 2 — Johnson et al. supplementary material [file S2732494X24003577sup002.docx]

**Supplemental Figures**

**Supplemental Figure 1:** **Re-visualization of the Adaptome Framework for Intervention Adaptation from Chambers & Norton (2016)**

**
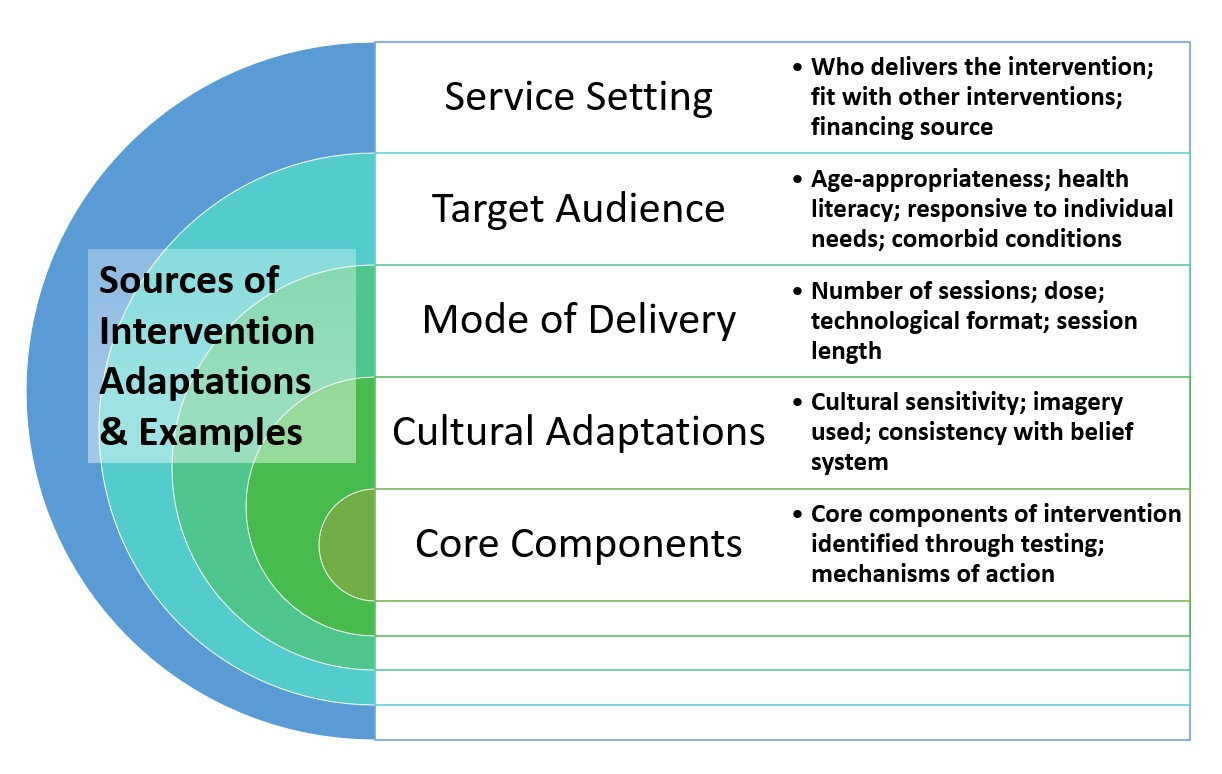
**

Permission to use and reference the Adaptome Framework was granted by the authors.

**Supplemental Figure 2: Example of Provider Prescribing Feedback Report Card**


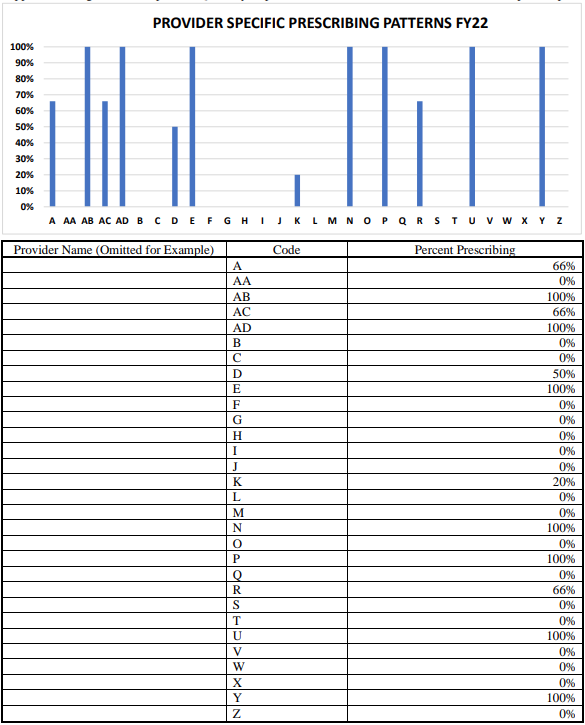


**Supplemental Figure 3: Monthly percentage of AUB/URI encounters with antibiotics with recalculation of control limits at each signal of special variation, Memphis**

**
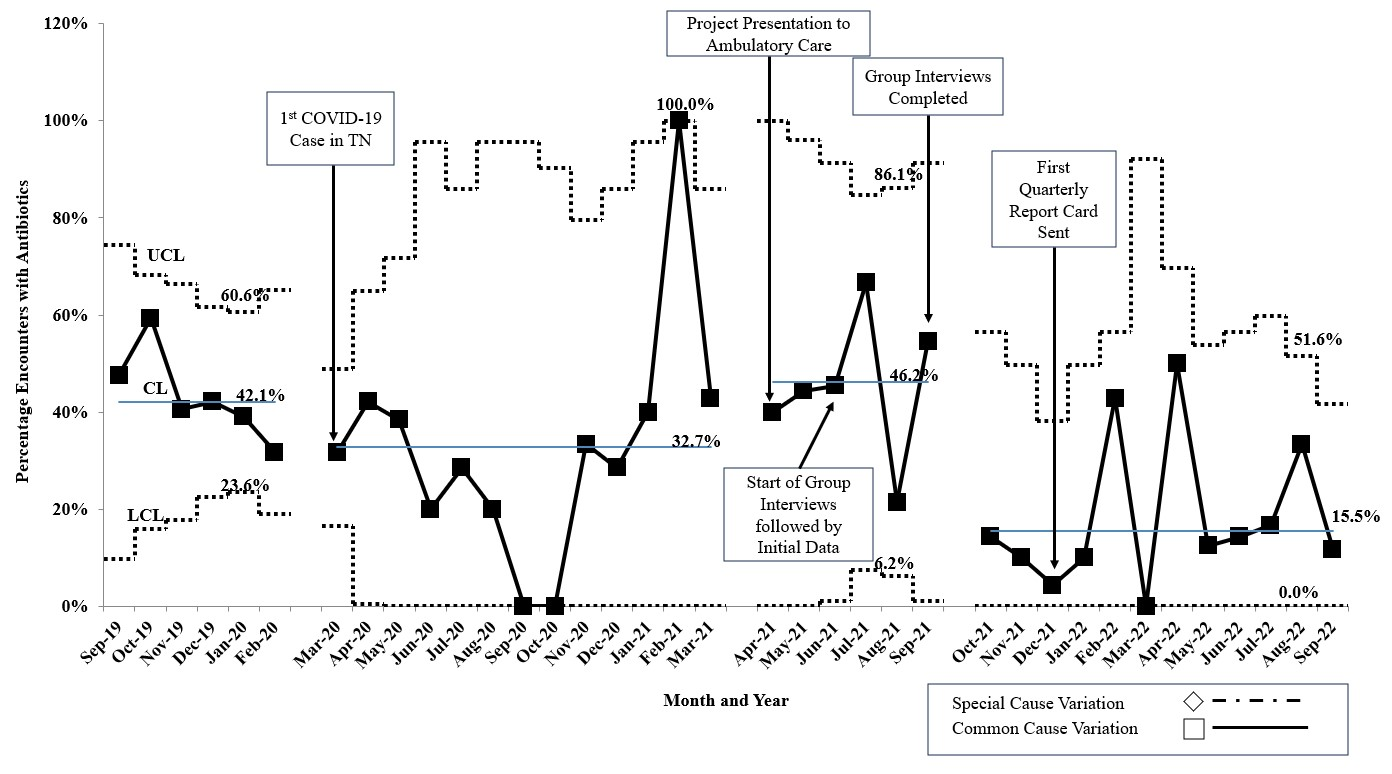
**

Recalculation of the control limits and mean with special cause variation noted April 2021 supports an unidentified force driving prescribing despite initiation of the interventions to reduce prescribing. However, from October 2021 through September 2022, after the special cause variation ends, there is return of common cause variation but the recalculated mean is significantly lower due to removal of the unexplained special cause variation and suggests a more substantial, sustained reduction in antibiotic prescribing from that reported in the primary analysis.

**Supplemental Figure 4:** **Monthly percentage of AUB/URI encounters with antibiotics with recalculation of control limits at each signal of special variation, TVHS**

**
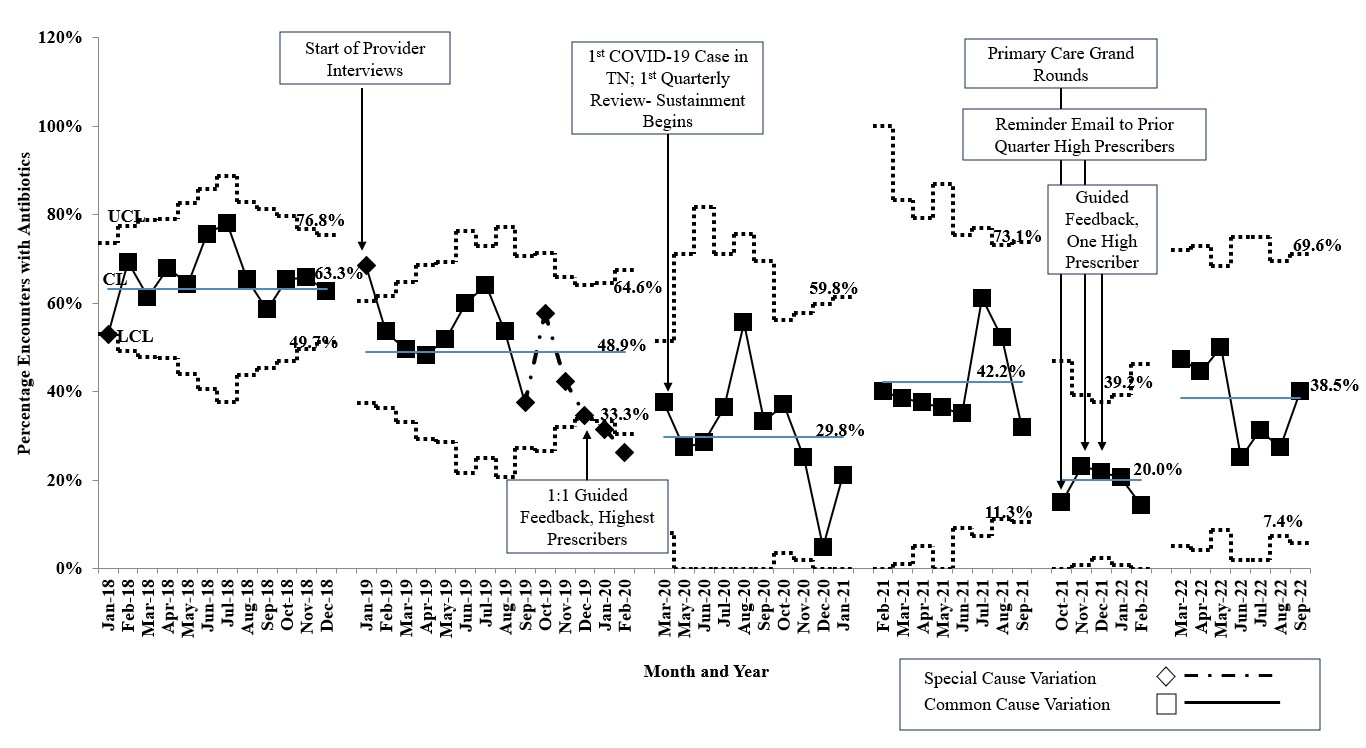
**

Due to COVID-19 logistic issues, April 2020 data was not collected and due to changes in how ICD-10 codes are extracted, could not be retrospectively pulled. After moving to sustainment in March 2020, there were two periods of special cause variation noted February 2021 through September 2021 and October 2021 through February 2022. It is not clear what drove the initial special cause variation with increased prescribing, but TVHS did add new providers who did increase prescribing in July and August 2021. Immediately thereafter, prescribing dropped suddenly and showed special cause variation with persistently lower prescribing. While interventions to lower prescribing were performed from October 2021 through December 2021, it is unclear what triggered the initial drop during that time. After prescribing returned to common cause variation, TVHS still showed overall reduced mean antibiotic prescribing of 38.5%, down from original 63.3%, but higher than the primary analysis mean of 31.7%, suggesting the impact was potentially less.
